# Acupuncture for symptomatic rotator cuff disease: Protocol for a systematic review and meta-analysis

**DOI:** 10.1097/MD.0000000000018716

**Published:** 2020-01-10

**Authors:** Seoyoung Choi, Kun Hyung Kim

**Affiliations:** a Department of Acupuncture & Moxibustion Medicine, Pusan National University Korean Medicine Hospital; b Division of Clinical Medicine, School of Korean Medicine, Pusan National University, Republic of Korea.

**Keywords:** acupuncture, protocol, rotator cuff disease, shoulder pain, systematic review

## Abstract

**Background:**

Rotator cuff disease (RCD) consists of subacromial impingement syndrome, rotator cuff tendinopathy or tendinitis, partial or full rotator cuff tear, calcific tendinitis, and subacromial bursitis. Acupuncture has been suggested as a meaningful nonsurgical intervention for managing shoulder pain and dysfunction. However, previous reviews have not completely addressed the role of acupuncture in the management of RCDs. The objective of this systematic review is to evaluate the effectiveness and safety of acupuncture for managing symptoms in patients with RCD.

**Methods:**

We will search the following databases from their inception to November 30, 2019: MEDLINE, EMBASE, Cochrane Central Register of Controlled Trials (CENTRAL), Cumulative Index to Nursing and Allied Health Literature (CINAHL), Allied and Complementary Medicine (AMED), Physiotherapy Evidence Database (PEDro), 3 Chinese databases (China Academic Journal Full-text Database (CAJ), China Doctoral Dissertations Full-text database and China Masters’ Thesis Full-text Database), 6 Korean databases (Korean studies Information Service System (KISS), National Digital Science Library (NDSL), Research Information Sharing Service (RISS), Korean Medical Database (KMBASE), Korea Institute of Science and Technology Information (KISTI), Oriental Medicine Advanced Searching Integrated System (OASIS)), and 3 trial registries (ClinicalTrials.gov, International Standard Randomized Controlled Trials Number (ISRCTN) Registry, WHO International Clinical Trials Registry Platform (ICTRP)). We will include randomized controlled trials of acupuncture for RCD. There will be no restrictions related to setting or location. The primary outcome is pain intensity and shoulder dysfunction measured on validated scales within 12 weeks. Quality assessment will be performed using the Cochrane risk of bias tool. Dichotomous outcomes will be presented as risk ratios (RR), and continuous outcomes will be presented as weighted or standardized mean differences (SMD). Each outcome will be calculated with 95% confidence intervals.

**Discussion:**

The updated evidence that would be provided by this review will offer useful information for patients and practitioners, and also have implications for future studies and the development of clinical practice guidelines of RCD.

Registration: Open Science Framework (OSF) Preregistration. 2019, December 5. osf.io/n2e6t

## Abstract 2

Supplemental Digital Content is available in the text

## 1 Introduction

Shoulder pain is one of the common musculoskeletal complaints, and significantly impacts a person's health-related quality of life. Rotator cuff diseases (RCDs) are the most common underlying cause of shoulder pain, with estimates of prevalence ranging from 65% to 85% depending on the context and characteristics of the study population.^[1]^ RCD is an umbrella term that refers to anatomical deformities, as well as symptoms and signs of rotator cuff pathologies, including subacromial impingement syndrome, rotator cuff tendinopathy or tendinitis, partial or full rotator cuff tear, calcific tendinitis, and subacromial bursitis.^[2,3]^ “Subacromial pain syndrome” is a comprehensive term, that describes non-traumatic shoulder problems with subacromial pain during abduction of the arm.^[4]^ RCD increases in prevalence with age and is more prevalent among participants who repetitively use arms in overhead motions (e.g., swimming and tennis).^[5]^

Although the exact mechanisms are unknown, the pathogenesis of RCD has been attributed to both intrinsic and extrinsic mechanisms. The intrinsic theory describes degeneration in the rotator cuff tendon itself as a primary cause, leading to impingement and a loss of structural integrity. It is argued that poor regenerative properties within a zone of hypovascularity and repetitive stresses, which result in inflammatory changes, could be significant factors in the degeneration process.^[2,6]^

The extrinsic theory hypothesizes that pressure damage occurs due to pathological contact between the rotator cuff and the acromion while lifting the arm. Extrinsic factors include those which affect the subacromial space, such as the shape of the acromion, the orientation of the scapula, and an alteration in the glenohumeral kinematics due to weakness in the rotator cuff.^[6,7]^

First line treatment options for RCD include nonoperative management. Some systematic reviews have reported on various nonoperative treatments such as physical therapy, exercise therapy, manual therapy, corticosteroid or hyaluronate injection, analgesics, non-steroidal anti-inflammatory drugs (NSAIDs), extracorporeal shockwave therapy, and acupuncture.^[8,9]^

Acupuncture is commonly used for musculoskeletal disorders, and it has been suggested as a meaningful nonsurgical intervention for managing shoulder pain and dysfunction.^[10]^ However, previous systematic reviews of acupuncture for shoulder pain included heterogeneous shoulder disorders that might respond to given treatments differently based on the underlying pathophysiology, and these reviews did not completely address the role of acupuncture in the management of RCDs. A wide range of acupuncture techniques as they are currently practiced was not fully reflected in the scope of the previous evidence, which implies an evidence-practice gap to be covered.^[9,11,12]^ The aim of the present systematic review is to assess the effectiveness and safety of a wide range of acupuncture techniques for managing symptoms in patients with RCD.

## 2 Methods

### 2.1 Study registration

Prospective registration of this study was approved by the Open Science Framework (OSF) registries (osf.io/n2e6t), and the protocol was written following the Preferred Reporting Items for Systematic Reviews and Meta-Analyses Protocols (PRISMA-P) statement guidelines^[13]^

### 2.2 Criteria for study selection

There will be no restrictions related to setting or location.

### 2.3 Types of studies

We will include randomized controlled trials (RCTs) of acupuncture treatment for rotator cuff disease. Quasi-randomized, non-randomized controlled trials, uncontrolled clinical trials (e.g., case studies), qualitative studies, and laboratory studies will be excluded. Crossover randomized trials, if any, will be included, but only the pre-crossover data will be analyzed in order to avoid carryover effects. Study eligibility will not be restricted by language or date of publication.

### 2.4 Types of participants

Our review will include all patients with a definite diagnosis or provisional diagnosis of RCD. We define a definite diagnosis as a diagnosis of RCD that has been confirmed by both clinical assessments and diagnostic imaging (e.g., ultrasound, magnetic resonance imaging). Patients with a provisional diagnosis of RCD include patients who are assumed to have RCD based solely on history taking and physical examinations without radiological assessment.

Patients with unspecified shoulder pain with or without radiological evidence of RCDs will be included only if eligibility criteria or patient characteristics are deemed to be compatible with a diagnosis of RCD.

If trials include a mixed population with shoulder diseases including RCD, we will try to retrieve the data for patients with RCD. If we do not succeed, we will exclude those trials.

We will exclude trials for patients having shoulder or arm pain due to systemic inflammatory conditions such as rheumatoid arthritis, fractures, adhesive capsulitis, osteoarthritis, hemiplegic shoulders, or myofascial pain of the upper extremity. Patients with RCD accompanied by myofascial pain of the upper extremity will be included, as myofascial pain is a common comorbid condition in patients with chronic shoulder pain. There will be no restriction on age, gender, and ethnic origin.

### 2.5 Types of interventions

For the purposes of this review, “acupuncture intervention” will be defined as:

A needle stimulation that elicits deqi sensation by penetrating the skin. There are no limitations on the methods of stimulation (e.g., electro-acupuncture, pharmacopuncture, thread-embedding therapy, acupotomy), points of stimulation (e.g., auricular points, scalp acupuncture points, tender points), types of needle, duration of treatment, or number of treatments.

When acupuncture was provided along with other active treatments for the acupuncture group, the same active treatment had to be given to control groups in order for the trial to be included. Acupoint-related interventions that do not penetrate the skin with needles (e.g., moxibustion, acupressure, or laser acupuncture) will be excluded.

Control interventions will include active treatments (e.g., oral medication, glucocorticoid injection, electrotherapy, manual therapy, or exercise), waiting list control or placebo treatment. Trials in which acupuncture was compared with other forms of acupuncture or acupoint stimulation will be excluded.

### 2.6 Types of outcome measures

We will collect information on all outcomes for all periods of time assessed in the included studies, and we will then categories them as short-term (up to 3 months post-randomization), intermediate-term (up to 6 months post-randomization), and long-term (more than 6 months post-randomization).

### 2.7 Primary outcomes

- Pain intensity measured on validated scales, such as the visual analogue scale (VAS) or numeric rating scale (NRS), within 12 weeks.
- Shoulder function measured on validated scales, such as the Constant-Murley score (CMS), Shoulder Pain and Disability Index (SPADI) total scale, Dutch Shoulder Disability Questionnaire (SDQ-NL), Disabilities of the Arm, Shoulder and Hand (DASH) score, University of California-Los Angeles (UCLA) Shoulder rating scale, or the American Shoulder and Elbow Surgeons (ASES) rating scale, within 12 weeks.

### 2.8 Secondary outcomes

- Pain intensity measured on validated scales, such as the VAS or NRS, over 12 weeks.
- Shoulder function measured on validated scales, such as the CMS, SPADI total scale, SDQ-NL, DASH score, UCLA Shoulder rating scale, or the ASES rating scale, over 12 weeks.
- Health-related quality of life measured on validated scales such as Short-Form 36 (SF-36) Health Survey or EuroQoL EQ-5D.
- Patient global assessment of treatment outcomes, such as the Patient Global Impression of Change (PGIC).
- Occurrence of adverse events.
- Active range of motion of shoulder joint.
- Muscle strength.
- Work disability, such as length or total days of sick leave.
- Proportion of patients who finally ended up receiving shoulder surgery.

### 2.9 Search methods for identification of studies

#### 2.9.1 Electronic searches

The following electronic databases will be searched from inception to November 30, 2019: MEDLINE, EMBASE, Cochrane Central Register of Controlled Trials (CENTRAL), Cumulative Index to Nursing and Allied Health Literature (CINAHL), Allied and Complementary Medicine (AMED), Physiotherapy Evidence Database (PEDro), 3 Chinese databases (China Academic Journal Full-text Database (CAJ), China Doctoral Dissertations Full-text database and China Masters’ Thesis Full-text Database), 6 Korean databases (Korean studies Information Service System (KISS), National Digital Science Library (NDSL), Research Information Sharing Service (RISS), Korean Medical Database (KMBASE), Korea Institute of Science and Technology Information (KISTI), Oriental Medicine Advanced Searching Integrated System (OASIS)), 3 trial registries (ClinicalTrials.gov, International Standard Randomized Controlled Trials Number (ISRCTN) Registry, WHO International Clinical Trials Registry Platform (ICTRP)).

### 2.10 Search for other resources

We will check the reference lists of the included studies and relevant reviews and, if necessary, contact researchers in the field of RCD to identify additional trials.

### 2.11 Search strategy

The search terms consist of 2 parts: rotator cuff disease (e.g., “rotator cuff,” “impingement,” “subacromial”) and acupuncture (e.g., “acupuncture,” “electroacupuncture,” “needling,” “pharmacopuncture,” “embedding therapy”, “acupotomy”). Combinations of Medical Subject Headings (MeSH) and text words will be used. Details of the search strategy for MEDLINE are available in Appendix.

### 2.12 Data collection and analysis

#### 2.12.1 Study selection

**Figure 1:**
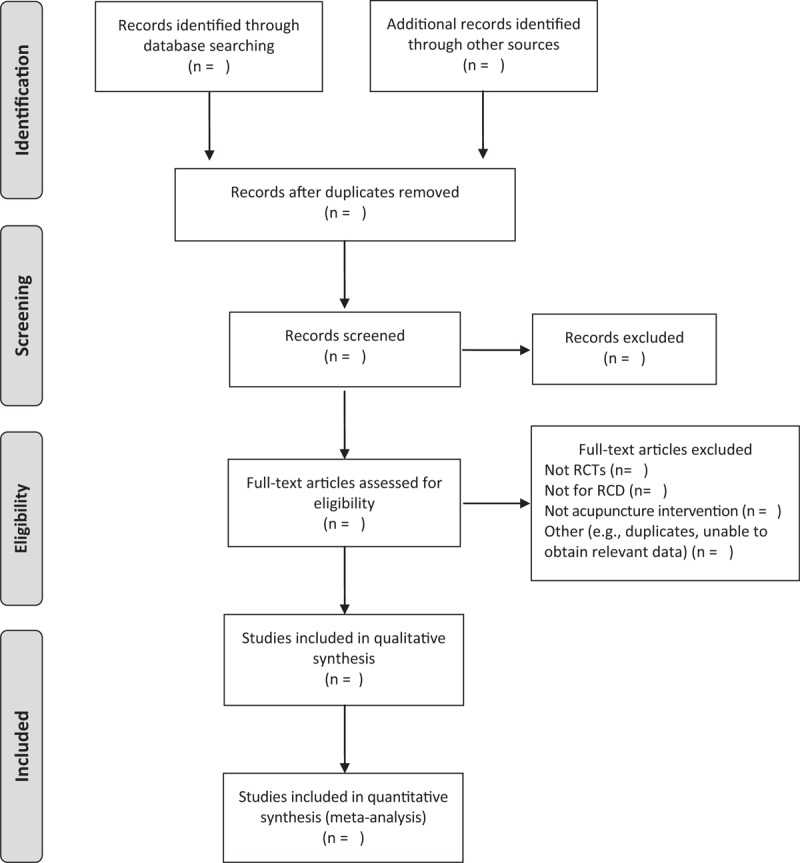
PRISMA flow diagram of study selection. RCT = randomized controlled trial, RCD = rotator cuff disease.

One review author will search potentially relevant studies. Two review authors will independently screen for titles and abstracts. After removing duplicates, they will then use the full-text articles to determine which papers will finally be included in the review. If the 2 review authors disagree about whether to include a paper, a third reviewer will decide on whether the paper will be included. The detailed process of study selection will be shown in a PRISMA flow diagram (Fig. 1).

#### 2.12.2 Data extraction and management

Two authors will independently extract relevant data from each of the eligible studies using a standardized data extraction form. The following information will be extracted: study characteristics (e.g., design, authors, date of publication, country, language, sample size), participants (e.g., age, gender, severity of symptoms at baseline), acupuncture and control intervention (e.g., number of sessions, duration, acupuncture points), outcome measures, results of intervention, duration of follow-up assessments, funding, conflict of interest, and reported adverse effects. We will extract details of the acupuncture treatment and control interventions on the basis of the revised Standards for Reporting Interventions in Clinical Trials of Acupuncture (STRICTA). We will contact the corresponding author to retrieve additional information if necessary.

### 2.13 Assessment of risk of bias in included studies

Two review authors will independently perform the assessment of risk of bias regarding 7 domains. We referred to the *Cochrane Handbook for Systematic Reviews of Interventions* for detailed description of the assessment of risk of bias.^[14]^ The 7 domains are as follows: random sequence generation, allocation concealment, blinding of participants, blinding of outcome assessors, incomplete outcome data, selective outcome reporting, and other sources of bias. For each item, risk of bias will be graded as high, low, or unclear.

Results of the quality assessment and rationale for the decisions will be presented in “risk of bias tables”, and will be used for grading the overall quality of evidence for each outcome.

### 2.14 Measures of treatment effect

Dichotomous outcomes will be presented as risk ratios (RR), and continuous outcomes will be presented as weighted or standardized mean differences (SMD). We will calculate 95% confidence intervals and two-sided *P* values for each outcome.

### 2.15 Unit of analysis issues

Multiple treatment groups in a trial will be combined into a single pairwise comparison. When the studies assessed outcome variables at multiple time points, we will categorize the time frames of the included studies into short-term (up to 3 months post-randomization), intermediate-term (up to 6 months post-randomization), and long-term (more than 6 months post-randomization). If there is more than 1 outcome measurement in the same time frame, we will extract the last measurement data within the time frame.

### 2.16 Dealing with missing data

We will try to contact the original investigators by email to request missing data. If the missing data were not obtained, we will analyze only the available data.

### 2.17 Assessment of heterogeneity

According to the *Cochrane Handbook for Systematic Reviews of Interventions*, assessment of between-trial heterogeneity will be based on visual inspection of the forest plot, and more formally on the *I*^*2*^ statistic. We define that an *I*^*2*^ of less than 40% is low, 30% to 60% is moderate, 50% to 90% is substantial, and 75% to 100% is considerable.^[14]^

### 2.18 Assessment of reporting bias

We will use funnel plots to evaluate reporting bias if we retrieve a minimum of 10 trials reporting the same outcome.

### 2.19 Data synthesis

A meta-analysis will be performed using RevMan software (Review Manager Version 5.3 for Windows, The Nordic Cochrane Centre, Copenhagen). A random effects model will be used to calculate the pooled effect estimates, because substantial clinical heterogeneity is expected among the studies included in this review. If considerable heterogeneity (*I*^*2*^ > 75%) is observed, we will not meta-analyze the trials and will qualitatively synthesize the data.

If an adequate number of studies are available, subgroup analysis will be performed to find possible sources of the heterogeneity. Classifications are as follows:

1. diagnosis of the study population (i.e., rotator cuff tear or without tear),
2. type of acupuncture stimulation (i.e., manual, electrical, or other stimulation techniques, such as pharmacoacupuncture, acupotomy, or thread-embedding therapy).

### 2.20 Sensitivity analysis

We will perform sensitivity analyses to determine whether the results have been influenced by trials where radiologic diagnosis of RCDs was not mentioned in the participant eligibility criteria, where measures of variance are missing, and where different methods of analysis were used (random-effects model or fixed-effect model).

### 2.21 Grading the quality of evidence

We will use the Grading of Recommendations Assessment, Development and Evaluation (GRADE) approach to assess the overall quality of the evidence for each main outcome with respect to the following domains: the risk of bias across studies, directness of evidence, heterogeneity, precision of effect estimates and risk of publication bias.^[14]^ Level of evidence will be classified into 4 categories: high, moderate, low, or very low.

### 2.22 Ethics and dissemination

Ethical approval is not necessary as this study will not require data from individual patients. The results of this review will be disseminated through peer-reviewed journal articles or conference presentations.

## 3 Discussion

There is evidence that supports the effectiveness and safety of acupuncture for chronic musculoskeletal conditions, including shoulder pain.^[15]^ However, it is still uncertain whether acupuncture is effective and safe for RCD, partially due to a lack of high quality primary studies and systematic reviews that specifically focus on RCD. Previous systematic reviews for chronic shoulder pain observed considerable heterogeneity associated with a wide range of different diagnoses, which provide, at best, limited evidence for the effectiveness of acupuncture for RCDs.^[11,12,16]^ Moreover, various acupuncture techniques, which are increasingly used in East Asian settings, are not addressed in previous reviews, which imply the necessity of both clinical trials and systematic reviews that assess the effectiveness and the safety of such interventions for patients with RCDs.^[9,11,12]^ Methodological limitations of previous reviews, including the exclusion of studies that had reported their findings in languages other than English^[12]^ or failure to register their reviews,^[9]^ also calls for systematic reviews addressing such problems. This systematic review would provide updated evidence of various types of acupuncture that specifically focuses on its effectiveness and safety for patients with RCDs.

## Author contributions

**Conceptualization:** Seoyoung Choi, Kun Hyung Kim.

**Funding acquisition:** Kun Hyung Kim.

**Methodology:** Seoyoung Choi, Kun Hyung Kim.

**Project administration:** Seoyoung Choi, Kun Hyung Kim.

**Writing – original draft:** Seoyoung Choi.

**Writing – review & editing:** Kun Hyung Kim.

Seoyoung Choi orcid: 0000-0003-0108-8617.

## Supplementary Material

Supplemental Digital Content

## References

[1] Karjalainen TV Jain NB Page CM Subacromial decompression surgery for rotator cuff disease. Cochrane Database Syst Rev 2019. 10.1002/14651858.CD005619.pub3 PMC6357907 30707445

[2] Lee YG Rotator cuff disease. In: The Korean Orthopaedic Association, ed. Orthopaedics. 7th edn Vol 1 2013;Seoul: ChoiSin Medical Publishing Co., 629–636.

[3] Page MJ Green S Mrocki MA Electrotherapy modalities for rotator cuff disease. Cochrane Database Syst Rev 2016 CD012225. 27283591 10.1002/14651858.CD012225 PMC8570637

[4] Diercks R Bron C Dorrestijn O Guideline for diagnosis and treatment of subacromial pain syndrome: a multidisciplinary review by the Dutch Orthopaedic Association. Acta Orthop 2014;85:314–22. 24847788 10.3109/17453674.2014.920991 PMC4062801

[5] Dang A Davies M Rotator Cuff Disease: Treatment Options and Considerations. Sports Med Arthrosc Rev 2018;26:129–33. 30059447 10.1097/JSA.0000000000000207

[6] Micallef J Pandya J Low AK Management of rotator cuff tears in the elderly population. Maturitas 2019;123:9–14. 31027684 10.1016/j.maturitas.2019.01.016

[7] Consigliere P Haddo O Levy O Subacromial impingement syndrome: management challenges. Orthop Res Rev 2018;10:83–91. 30774463 10.2147/ORR.S157864 PMC6376459

[8] Jancuska J Matthews J Miller T A systematic summary of systematic reviews on the topic of the rotator cuff. Orthop J Sports Med 2018;6:2325967118797891. 30320144 10.1177/2325967118797891 PMC6154263

[9] Choi HM Han SY Hwang DR Acupuncture treatment for rotator cuff disorder: a systematic review. J Korean Med Rehab 2018;28:11–20.

[10] Birch S Lee MS Alraek T Overview of treatment guidelines and clinical practical guidelines that recommend the use of acupuncture: a bibliometric analysis. J Altern Complement Med 2018;24:752–69. 29912569 10.1089/acm.2018.0092

[11] Green S Buchbinder R Hetrick SE Acupuncture for shoulder pain. Cochrane Database Syst Rev 2005;2:CD005319. 10.1002/14651858.CD005319 PMC12130916 15846753

[12] Hall ML Mackie AC Ribeiro DC Effects of dry needling trigger point therapy in the shoulder region on patients with upper extremity pain and dysfunction: a systematic review with meta-analysis. Physiotherapy 2018;104:167–77. 29439829 10.1016/j.physio.2017.08.001

[13] Shamseer L Moher D Clarke M Preferred reporting items for systematic review and meta-analysis protocols (PRISMA-P) 2015: elaboration and explanation. BMJ 2015;349:g7647. 10.1136/bmj.g7647 25555855

[14] Higgins JPT Green S Cochrane handbook for systematic reviews of interventions. Chichester, West Sussex; Hoboken NJ: John Wiley & Sons; 2008.

[15] Vickers AJ Vertosick EA Lewith G Acupuncture for chronic pain: update of an individual patient data meta-analysis. J Pain 2018;19:455–74. 29198932 10.1016/j.jpain.2017.11.005 PMC5927830

[16] Cox J Varatharajan S Côté P Optima Collaboration. Effectiveness of acupuncture therapies to manage musculoskeletal disorders of the extremities: a systematic review. J Orthop Sports Phys Ther 2016;46:409–29. 27117725 10.2519/jospt.2016.6270

