# Supplementary material for: Acupuncture for symptomatic rotator cuff disease: Protocol for a systematic review and meta-analysis
Source: Medicine (Baltimore). 2020 Jan 10;99(2):e18716. doi: 10.1097/MD.0000000000018716 (PMC6959934; doi:10.1097/MD.0000000000018716)
Supplement: Supplemental Digital Content [file medi-99-e18716-s001.docx]

**Appendix. Detailed search strategy for MEDLINE**

#1 "Rotator Cuff"[Mesh] OR "shoulder"[Title/Abstract] OR "rotator cuff"[Title/Abstract] OR “subacromial”[Title/Abstract] OR "supraspinatus"[Title/Abstract] OR "infraspinatus"[Title/Abstract] OR "teres minor"[Title/Abstract] OR "subscapularis"[Title/Abstract]

#2 "Rotator Cuff Tear Arthropathy"[Mesh] OR "Rotator Cuff Injuries"[Mesh] OR "Shoulder Impingement Syndrome"[Mesh] OR “Shoulder Pain”[Mesh] OR "Bursitis"[Mesh] OR “bursitis”[Title/Abstract] OR “impingement”[Title/Abstract] OR “tendinopathy”[Title/Abstract] OR “tendinitis”[Title/Abstract] OR “tendonitis”[Title/Abstract] OR “tear”[Title/Abstract] OR “rupture”[Title/Abstract] OR “tenosynovitis” [Title/Abstract]

#3 #1 AND #2

#4 “Acupuncture”[Mesh] OR “Acupuncture Therapy”[Mesh] OR “Acupuncture Points”[Mesh] OR “Meridians”[Mesh] OR “Acupuncture”[Title/Abstract] OR “needl*”[Title/Abstract]

#5 “Electroacupuncture”[Mesh] OR “electroacupuncture”[Title/Abstract] OR “electro-acupuncture”[Title/Abstract]

#6 “Trigger Points”[Mesh]

#7 “Acupuncture, Ear”[Mesh] OR “Auriculotherapy”[Mesh] OR “auricular acupuncture”[Title/Abstract] OR “ ear acupuncture”[Title/Abstract] OR “auricular needl*”[Title/Abstract] OR “ear needl*”[Title/Abstract]

#8 “pharmacopuncture”[Title/Abstract] OR “pharmacoacupuncture”[Title/Abstract] OR “pharmaco-acupuncture”[Title/Abstract] OR “acupoint injection”[Title/Abstract]

#9 “fire acupuncture”[Title/Abstract] OR “fire needl*”[Title/Abstract] OR “warm acupuncture”[Title/Abstract] OR “warm needl*”[Title/Abstract]

#10 “scalp acupuncture”[Title/Abstract]

#11 “thread embedding”[Title/Abstract] OR “catgut embedding”[Title/Abstract] OR “needle embedding”[Title/Abstract] OR “embedding acupuncture”[Title/Abstract]

#12 “acupotomy”[Title/Abstract] OR “needle knife”[Title/Abstract]

#13 OR/#4-#12

#14 #3 AND #13
